# Delayed hypercoagulable state in COVID‐19 adolescent patient: a case report

**DOI:** 10.1002/rcr2.793

**Published:** 2021-06-08

**Authors:** Desdiani Desdiani, Nita Yulianti, Anindita Basuki

**Affiliations:** ^1^ Faculty of Medicine Universitas Sultan Ageng Tirtayasa Cilegon Indonesia; ^2^ Department of Pulmonology and Respiratory Medicine Bhayangkara Brimob Hospital Depok Indonesia; ^3^ Department of Clinical Pathology Bhayangkara Brimob Hospital Depok Indonesia; ^4^ Department of Radiology Bhayangkara Brimob Hospital Depok Indonesia

**Keywords:** Adolescent patient, COVID‐19, hypercoagulation

## Abstract

Coronavirus disease 2019 (COVID‐19) is a systemic hyperinflammation disease which can cause severe respiratory symptoms and extrapulmonary manifestations. Hypercoagulable state in COVID‐19 adolescent patient is a rare case. We present the case of a 16‐year‐old Indonesian boy with mild COVID‐19 symptoms. Initially, the patient was treated with azithromycin, N‐acetyl cysteine, etc. After several days of the treatment, there was clinical improvement. However, on day 15, the patient experienced hypercoagulation and stroke‐like symptoms. The patient was then subjected to additional drugs, including low‐molecular weight heparin (LMWH), and peripheral neuropathy vitamin therapy. On day 20, the clinical symptoms reduced. This case demonstrates the need for further study of the association between COVID‐19 and stroke in young population and the use of anticoagulants to prevent thrombotic events.

## Graphical abstract

We report a case of an adolescent patient with mild symptoms of coronavirus disease 2019 (COVID‐19) who was already in recovery state, but suddenly experienced hypercoagulable state and stroke‐like symptoms.

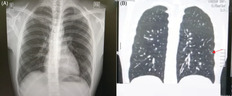

## Introduction

Coronavirus disease 2019 (COVID‐19) is a disease which can cause systemic hyperinflammation and has spread worldwide. The usual symptoms are fever, cough, malaise, anosmia, loss of appetite, diarrhoea, and other varying symptoms [1]. To date, there is no definitive treatment which can cure the disease. Elderly patients or patients with existing comorbidities have increased risks of severe illness from COVID‐19. Most of the reported cases are those with some complications, such as hypercoagulation. Some cases reported that this condition could occur in young patients when the potency of virus infection has started to decrease. Endothelial injury due to COVID‐19 can trigger hypercoagulable state. We report a case of an adolescent patient with mild symptoms of COVID‐19 who was already in recovery state, but suddenly experienced hypercoagulable state and stroke‐like symptoms.

## Case Report

**Figure 1:**
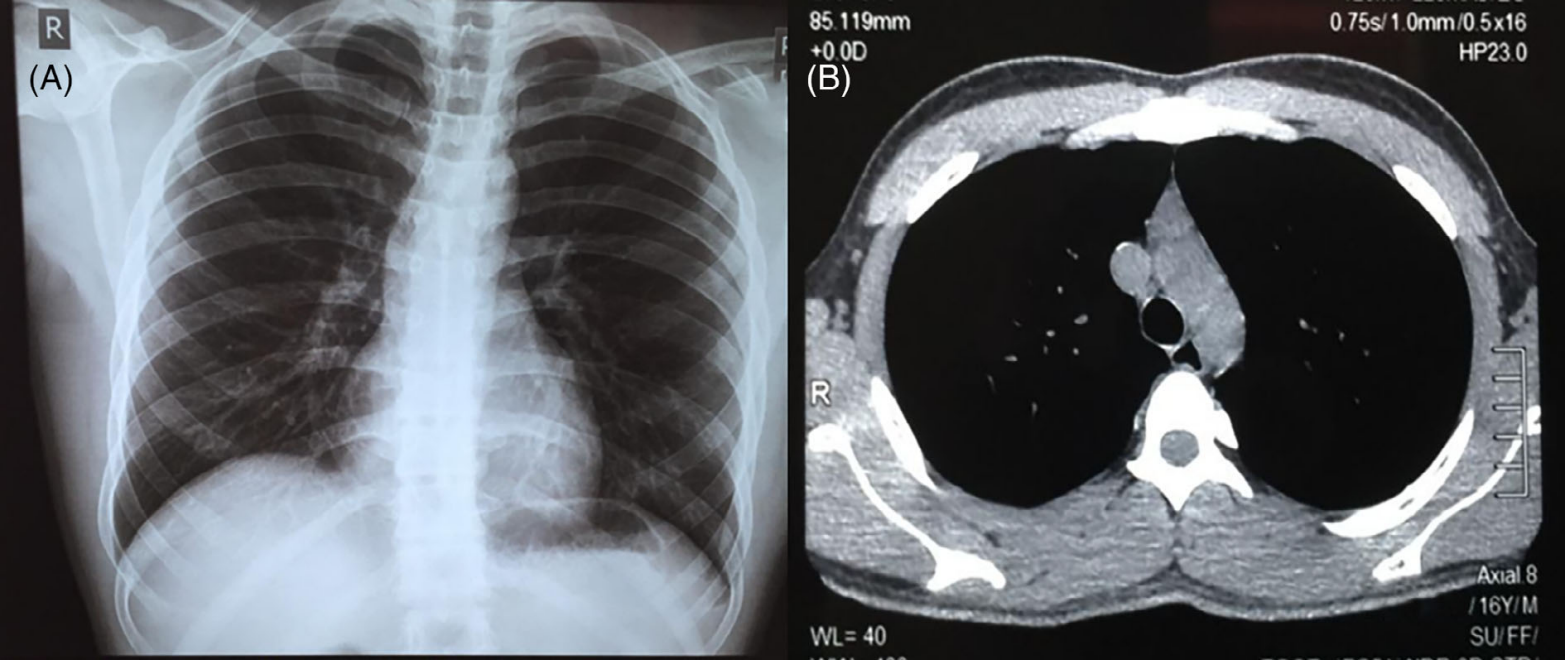
Chest imaging. (A) Chest radiography showing no radiological abnormalities both in the heart and lungs. (B) Computed tomography (CT) scan of the chest at that time showed no visible picture of acute lung inflammation, lung mass, or mediastinal tumour.

A 16‐year‐old Indonesian boy with fever and cough, and a history of close contact with a confirmed case COVID‐19 patient was admitted to the emergency department in our hospital. From anamnesis, there was no comorbidity (e.g. hypertension, diabetes mellitus, autoimmune disease, or malignancy). Vital signs and oxygen saturation were in normal ranges. Laboratory, radiology, and nasopharyngeal swab polymerase chain reaction (PCR) tests were conducted and the results were positive for COVID‐19 with CT values of RdrP: 28.05, E: 27.08. Laboratory examination showed leucocytes of 3720 cells/μL, platelet 241,000 cells/μL, lymphocytes 10%, monocytes 13%, neutrophil‐to‐lymphocyte ratio (NLR) 7.1, absolute lymphocyte count (ALC) 780 μL, d‐dimer 269 ng/mL, quantitative C‐reactive protein (CRP) < 5 mg/L, prothrombin time 13.1 sec, the international normalized ratio (INR) 0.96, and partial thromboplastin of time 23.7. The posteroanterior (PA) chest X‐ray showed no radiological abnormalities in the heart and lungs (Fig. 1A). Computed tomography (CT) scan of the chest at the time also showed no visible acute lung inflammation, lung mass, or mediastinal tumour (Fig. 1B). The patient was treated with azithromycin, N‐acetyl cysteine, paracetamol, and other supporting medications such as zinc, vitamin D3, vitamin C, and curcumin.

After several days of the treatment, the patient showed clinical improvement. On day 13, the patient had a nasopharyngeal swab PCR examination with a positive result of CT values of RdrP: 33.79, E: 33.77. On day 15 of the treatment, the patient suddenly had a high fever accompanied by severe and throbbing headaches, flatulence, nausea and vomiting, abdominal pain, numbness of arms, tingling and immobility of legs, and oxygen saturation of 93%.

**Figure 2:**
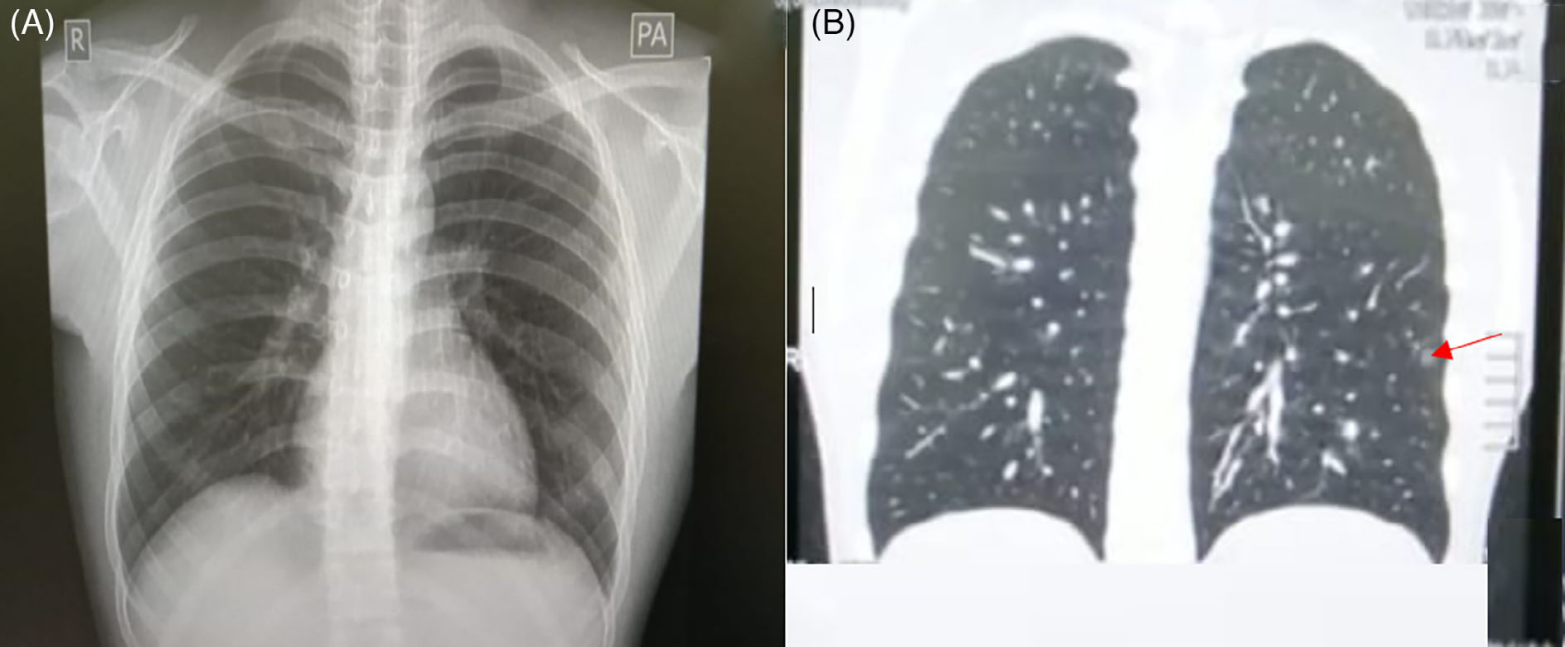
Chest imaging. (A) Chest radiography showing no radiological abnormalities both in the heart and lungs. (B) Computed tomography (CT) scan of the chest showed a solitary nodular ground‐glass opacity in S6 left lung (red arrow).

The patient was then subjected to additional drugs, such as meropenem, dexamethasone, remdesivir, low‐molecular weight heparin (LMWH) for five days, ondansetron, omeprazole, and supplements (e.g. vitamin C, zinc, and vitamin D3). Laboratory examination showed leucocytes of 12,220 cells/μL, platelet 260,000 cell/μL, monocytes 12%, NLR 17.72, lymphocytes 4.4%, d‐dimer 16,180 ng/mL, quantitative CRP 26.4 mg/L, activated partial thromboplastin time (APTT) 19.9 sec, potassium 3.2, and aspartate aminotransferase (AST) of 51 U/L. Radiology examination showed no radiological abnormalities both in the heart and lungs (Fig. 2A). CT scan of the chest showed solitary ground‐glass opacity nodules on S6 left lung (Fig. 2B). We consulted a neurologist, and the patient was then given additional peripheral neuropathy vitamin therapy such as methylcobalamin and vitamin B1 for five days. Furthermore, the patient was also given oxygen therapy and he felt better after that.

On day 20 of the treatment, the clinical symptoms reduced. The patient was able to move the limbs again and the tingling sensation reduced. The d‐dimer value of 460 ng/mL, quantitative CRP of 6.1 mg/L, lymphocytes 13%, platelet 284,000 cell/μL and the other parameters were within normal ranges, although the PCR result was still positive. At the end of the third week, the patient was discharged from the hospital. The patient's PCR result was negative. The laboratory and radiology results had also improved.

## Discussion

Pisano et al. reported an African‐American 33‐year‐old female COVID‐19 patient with acute malignant middle cerebral artery infarction. The severe acute respiratory syndrome coronavirus 2 (SARS‐CoV‐2) was reported to cause a thrombotic event [2]. COVID‐19 patients might experience thrombocytopenia, prolonged prothrombin time, increased fibrinogen, and d‐dimers [3]. Other markers of coagulation and inflammation that can be abnormal include ferritin, von Willebrand factor (VWF), CRP, complement, and cytokines. Thrombotic events occur in one‐third of COVID‐19 patients which are dominated by pulmonary embolism and associated with the severity of disease and increased mortality [4]. A study of five patients (younger than 50 years) infected with COVID‐19 who did not have risk factors for vascular diseases and were hospitalized with symptoms such as stroke showed that these cases have increased sevenfold compared with the previous year and were associated with COVID‐19. The patient's laboratory results in our case showed a hypercoagulable state, leading to the postulation that stroke observed in the young patient may be associated with SARS‐CoV‐2. This patient had never previously received vaccines and heparin. The clinical status of COVID‐19 patients with hypercoagulable state and stroke‐like symptoms was worse than non‐COVID‐19 stroke patients because it is related to the pathophysiology of the COVID‐19 [5].

To date, data which support the association between COVID‐19 and stroke in young population without specific risk factors (sometimes only mild respiratory symptoms) are increasing. Further studies are necessary to investigate this association and the use of anticoagulants to prevent thrombotic events. Our case underlines young patients with hypercoagulation and stroke symptoms but not yet confirmed with COVID‐19 need to be thoroughly investigated, including the patients with mild COVID‐19 symptoms, especially if new neurological symptoms arise.

### Disclosure Statement

Appropriate written informed consent was obtained for publication of this case report and accompanying images.

### Author Contribution Statement

Desdiani Desdiani: Conception, acquisition of information, analysis or interpretation data, drafting the manuscript, and final approval of the version to be published. Nita Yulianti: Analysis or interpretation of laboratory data. Anindita Basuki: Analysis or interpretation of radiography data.
